# Significant predictive factors of the severity and outcomes of the first attack of acute angioedema in children

**DOI:** 10.1186/s12887-019-1809-8

**Published:** 2019-11-11

**Authors:** Yuan-Jhen Syue, Chao-Jui Li, Wen-Liang Chen, Tsung-Han Lee, Cheng-Chieh Huang, Mei-Chueh Yang, Chih-Ming Lin, Meng-Huan Wu, Chu-Chung Chou, Chin-Fu Chang, Yan-Ren Lin

**Affiliations:** 1 Department of Anesthesiology, Kaohsiung Chang Gung Memorial Hospital, Chang Gung University College of Medicine, Kaohsiung, Taiwan; 2 Department of Emergency Medicine, Kaohsiung Chang Gung Memorial Hospital, Chang Gung University College of Medicine, Kaohsiung, Taiwan; 3 Department of Leisure and Sports Management, Cheng Shiu University, Kaohsiung, Taiwan; 4 Department of Biological Science and Technology, National Chiao Tung University, Hsinchu, Taiwan; 5 Department of Emergency Medicine, Changhua Christian Hospital, 135 Nanshsiao Street, Changhua, 500 Taiwan; 6 Department of Neurology, Changhua Christian Hospital, Changhua City, Taiwan; 7 Department of Social Work and Child Welfare, Providence University, Taichung, Taiwan; 8 Department of Medicinal Botanicals and Health Applications, Da-Yeh University, Changhua County, Taiwan; 9 School of Medicine, Chung Shan Medical University, Taichung, Taiwan; 10 School of Medicine, Kaohsiung Medical University, Kaohsiung, Taiwan

**Keywords:** Angioedema, Children, First attack, Urticaria, Allergy, Epinephrine

## Abstract

**Background:**

The initial episode of angioedema in children can be potential life-threatening due to the lack of prompt identification and treatment. We aimed to analyze the factors predicting the severity and outcomes of the first attack of acute angioedema in children.

**Methods:**

This was a retrospective study with 406 children (< 18 years) who presented in the emergency department (ED) with an initial episode of acute angioedema and who had subsequent follow-up visits in the out-patient department from January 2008 to December 2014. The severity of the acute angioedema was categorized as severe (requiring hospital admission), moderate (requiring a stay in the short-term pediatric observation unit [POU]), or mild (discharged directly from the ED). The associations among the disease severity, patient demographics and clinical presentation were analyzed.

**Result:**

In total, 109 (26.8%) children had severe angioedema, and the majority of those children were male (65.1%). Most of the children were of preschool age (56.4%), and only 6.4% were adolescents. The co-occurrence of pyrexia or urticaria, etiologies of the angioedema related to medications or infections, the presence of respiratory symptoms, and a history of allergies (asthma, allergic rhinitis) were predictors of severe angioedema (all *p* < 0.05). Finally, the duration of angioedema was significantly shorter in children who had received short-term POU treatment (2.1 ± 1.1 days) than in those who discharged from ED directly (2.3 ± 1.4 days) and admitted to the hospital (3.5 ± 2.0 days) (*p* < 0.001).

**Conclusion:**

The co-occurrence of pyrexia or urticaria, etiologies related to medications or infections, the presence of respiratory symptoms, and a history of allergies were predictors of severe angioedema. More importantly, short-term POU observation and prompt treatment might be benefit for patients who did not require hospital admission.

## Background

Acute angioedema is swelling due to fluid leaking from blood vessels into the surrounding dermis and subcutaneous layers. There are multiple mechanisms by which angioedema occurs [1–3]. Allergic angioedema (histamine-mediated) is the most common type [4–6]. Allergic angioedema has recognizable triggers, such as an insect bites or stings, food, or medication, and rapid onset of swelling [2, 7, 8]. Non-allergic, drug-induced angioedema is caused by certain medicines, such as angiotensin-converting enzyme inhibitors (ACEI) [9–12]. Among patients with allergic angioedema, the most common anatomical areas involved are around the eyes, lips, mouth, tongue, extremities, and genitalia [5, 6, 13]. An itchy, raised rash called urticaria can occur with angioedema. Angioedema may also lead to abdominal pain due to the swelling of the intestinal tract [14–16].

Sometimes, more severe symptoms occur, including swelling of the airway, which leads to suffocation, hypoxia and even respiratory failure [4, 17, 18]. Although the attack of angioedema might be easily aware (i.e., sudden swelling of eyelid or lip). However, for some parents, the severity of angioedema (life threatening or not) is not easily determined by themselves, especially when their children are suffering their first episodes. Furthermore, due to the lack of adequate self-identification and, therefore, the lack of a quick response, the first episode is potentially life threatening. Some studies have reported the demographics of patients with angioedema and the etiologies of angioedema, but the factors that predict life-threatening angioedema in children are still unclear. The purpose of the study was to clarify the predictors of the severity of the first attack of angioedema in children.

## Methods

### Patient population

During the period from January 1, 2008, to December 31, 2014, acute angioedema was diagnosed in 525 children aged less than 18 years at a 2500-bed medical center in central Taiwan. Among those patients, 483 children underwent medical treatment in the pediatric ED, and 429 of those children were identified as having their first episode of acute angioedema. A first attack of acute angioedema was defined as angioedema symptoms presenting for less than 6 weeks in patients without any history of angioedema. Of the 429 children who presented with an initial episode of acute angioedema, 20 were excluded from the study because they were lost to follow-up. Three children were excluded due to having hereditary angioedema. Therefore, the final study population was composed of 406 patients. The study protocol was approved by the Institutional Review Board (IRB) of the study hospital.

### Study design

Patient characteristics were obtained from hospital chart records and included the date of disease onset, age at onset, gender, possible etiologies, major clinical findings, co-occurrence of urticaria, co-occurrence of pyrexia, co-occurrence of anaphylactic shock, sites of angioedema, personal allergic history, treatments, and total symptom duration of angioedema. The children were divided into the following four age groups: infant (1 month-1 year), preschool age (2–6 years), school age (7–12 years), and adolescent (13–18 years). The possible etiologies were divided into the following seven major categories: (1) medications, (2) foods, (3) various infections, (4) inhalants (inhalation allergens, including animal dander, cockroach, dust mites, pollen), (5) insect bites and stings, (6) contact materials (contact allergens, angioedema was induced by skin contact with something), and (7) unknown causes. The relationships between these possible etiologies and a first attack of acute angioedema were determined by clinician review of the patients’ medical histories and clinical assessments. The possible etiologies of acute angioedema were determined based on statements made by the patients or their family members about particular life events or environmental exposure. For example, either viral or bacterial infection was suspected as the possible etiology when patients presented with angioedema after a recent bacterial or viral infection. Food or medications were suspected to be the possible etiologies when patients or family members stated that they suffered from skin symptoms after eating a particular food or taking a particular medication. Because infections and medications usually occurred in conjunction with each other, or with short intervening periods, clearly differentiation between those two etiologies was challenging. We used the following two methods to help distinguish between infection and medication as the cause of the angioedema: (1) determining whether the patient had previously taken the medication without experiencing an allergic response before it was prescribed to treat this infection, which implied the etiology was likely to be the infection, and (2) checking the adverse drug reaction (ADR) records from their medical records or insurance cards. Unknown causes were suspected when clinical assessments revealed nonspecific findings, and patients or family members denied any related life events or exposure to environmental stressors such as changes in diet, recent medication use, or contact with animals, plants or specific materials.

The major clinical findings in children with a first attack of acute angioedema included the following six groups of constitutional symptoms: (1) skin lesions, (2) respiratory tract symptoms (dyspnea, cough, rhinorrhea, sore throat, shortness of breath), (3) cardiovascular symptoms (unstable blood pressure, cardiac dysrhythmia), (4) neurological symptoms (dizziness, vertigo, convulsions, headache, changes in consciousness), (5) gastrointestinal symptoms (nausea, vomiting, diarrhea, constipation, abdominal pain), and (6) multiple (two or more groups of symptoms). The personal allergic history of the patients, including asthma, allergic rhinitis was obtained from hospital chart records and statements from patients or family members. The types of medical treatment that improved symptoms were recorded. Antihistamines, either H1-antagonists or H2-antagonists, and corticosteroids were used to treat angioedema in all patients. Epinephrine was used in children who presented with obvious respiratory distress or anaphylactic shock [4, 18, 19]. The treating physicians were all pediatric emergency medicine specialists. Dosages of medications were based on body weight in all cases. Medications were administered via injection only in children who required hospitalization or further observation in the pediatric observation unit (POU). The POU is designed for children who do not require inpatient admission but need to stay in the hospital for further observation and short-term treatment. The patients come directly to the POU from the pediatric ED or outpatient department (OPD) with a set of admission orders and admission notes. Once in the POU, these patients are evaluated, and the orders are reviewed by physicians. Patients are discharged from the POU when they are clinically stable. Patients who do not become clinically stable during observation are admitted as inpatients. The total duration of angioedema was defined as the period from the onset of symptoms to the resolution of symptoms. The information on the duration of angioedema was obtained from descriptions recorded by physicians during follow-up in the ED or OPD and from statements provided by patients or family members. The factors associated with the total duration of angioedema were analyzed.

In this study, the severity of acute angioedema was categorized according to the needs of hospitalization (angioedema was the primary diagnosis/major reason for admission). The severities could be divided into severe (requiring hospital admission,), moderate (requiring short-term POU observation), or mild (discharged directly from ED) according to the clinical presentations of the patients and the relevant medical treatments. Variables that could be related to the development of mild, moderate, and severe angioedema were analyzed to determine the predictors of the severity of disease.

### Statistical analysis

Data were analyzed by one-way ANOVA and the chi-square tests. The results of the descriptive analyses of the independent variables (age, gender, possible etiologies, clinical presentations, co-occurrence of other conditions, sites of angioedema, personal allergic histories, and treatments) are reported as percentages and means ± standard deviation (S.D.). Factors that might be associated with the severity of a first attack of acute angioedema (mild, moderate and severe) and the relationships between asthma and hospitalization (POU or hospital admission) were both analyzed by the Chi-Square test. The relationships among possible etiologies, treatments, and the duration of urticaria were analyzed by one-way ANOVA. A *P*-value less than 0.05 was regarded as significant. All analyses were performed with SPSS for Windows (Version 15.0, SPSS Inc., Chicago, IL).

## Result

### Demographics, etiologies, clinical presentations and treatments

**Fig. 1:**
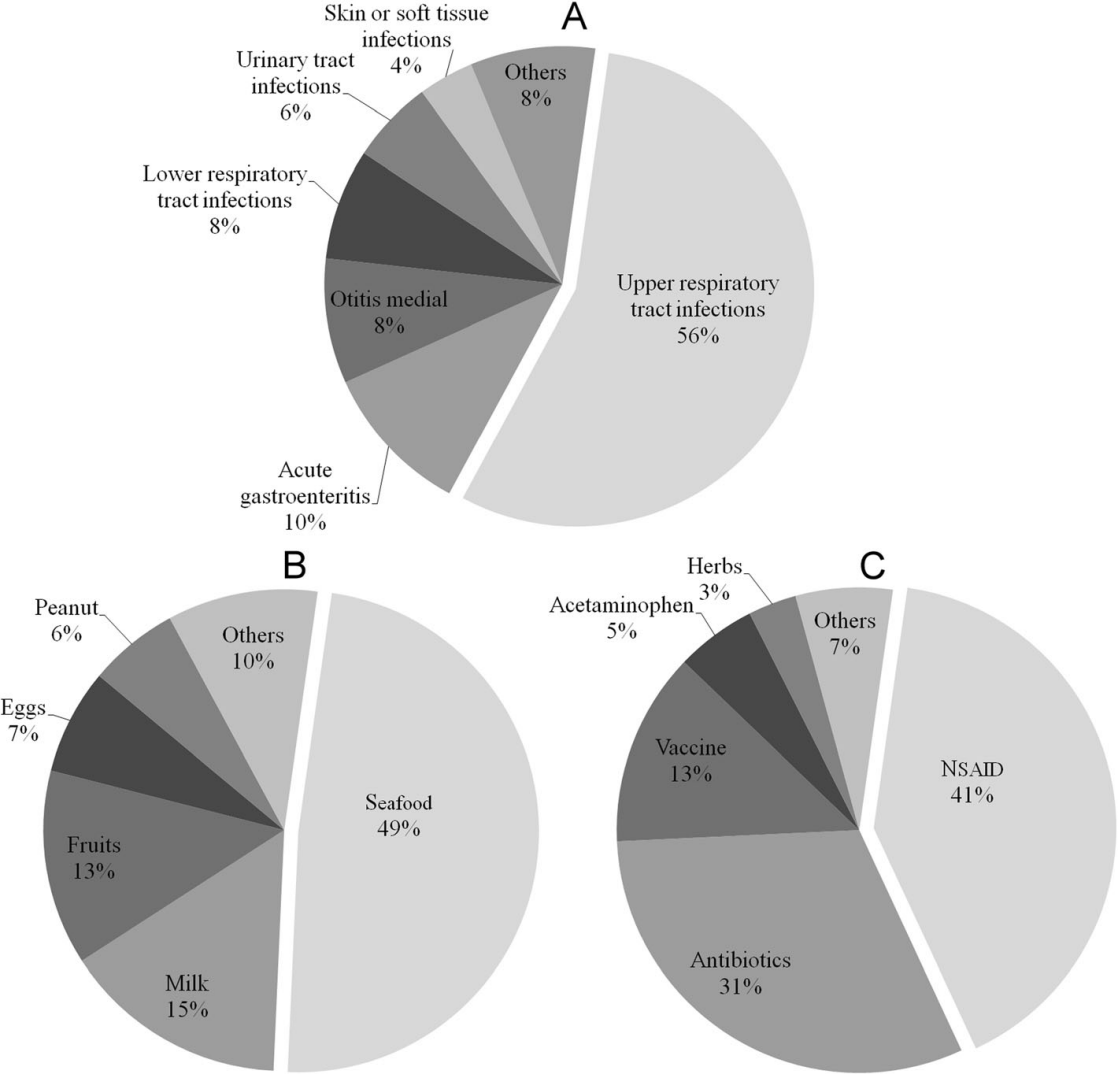
Detailed information on the three most common etiologies of the first attacks of angioedema in children. **a** Various infections (*n* = 103, 25.4%) were the most common possible etiology followed by (**b**) foods (*n* = 99, 24.4%) and (**c**) medications (*n* = 93, 22.9%)

This study included a total of 406 children aged ≤18 years (mean age, 6.4 ± 4.5 years) who experienced a first attack of acute angioedema. All children were followed in the ED or OPD during the study period. Their demographic information is shown in Table 1. Various infections (25.4%) were the most common possible etiologies, followed by food (24.4%) and medication (22.9%). (Detailed information on these three etiologies is shown in Fig. 1.) Only 2 (0.5%) children suffered angiotensin converting enzyme inhibitors (ACEI) related angioedema.

**Table 1:** Demographics and predictors of the severity of angioedema in children

Variables	Children with a first attack of angioedema (*N* = 406)				*p*-value
	All	Mild angioedema (ED discharge) *N* = 200	Moderate angioedema (POU observation) *N* = 97	Severe angioedema (Hospital admission) *N* = 109	
	No. (%)	No. (%)	No. (%)	No. (%)	
Gender^a^					
Male	242 (59.6)	107 (53.5)	64 (66.0)	71 (65.1)	0.047
Female	164 (40.4)	93 (46.5)	33 (34.0)	38 (34.9)	
Age^a^					
Infant	64 (15.8)	40 (20.0)	6 (6.2)	18 (16.5)	< 0.001
Preschool age	193 (47.5)	93 (46.5)	38 (39.2)	62 (56.9)	
School age	91 (22.4)	39 (19.5)	30 (30.9)	22 (20.2)	
Adolescent	58 (14.3)	28 (14.0)	23 (23.7)	7 (6.4)	
Possible etiologies of angioedema^a^					
Medications	93 (22.9)	35 (17.5)	37 (38.1)	21 (19.3)	< 0.001
Foods	99 (24.4)	52 (26.0)	28 (28.9)	19 (17.4)	
Various infections	103 (25.4)	42 (21.0)	20 (20.6)	41 (37.6)	
Inhalants	6 (1.5)	4 (2.0)	2 (2.1)	0 (0)	
Insects bites and stings	12 (3.0)	2 (1.0)	0 (0)	10 (9.2)	
Contact materials	2 (0.5)	0 (0)	2 (2.1)	0 (0)	
Unknown causes	91 (22.4)	65 (32.5)	8 (8.2)	18 (16.5)	
Clinical presentations^a^					
Only skin lesions	143 (35.2)	87 (43.5)	39 (40.2)	17 (15.6)	< 0.001
Respiratory tract symptoms	165 (40.6)	76 (38.0)	34 (35.1)	55 (50.5)	
Cardiovascular symptoms	28 (6.9)	6 (3.0)	8 (8.2)	14 (12.8)	
Neurological symptoms	11 (2.7)	6 (3.0)	2 (2.1)	3 (2.8)	
Gastrointestinal symptoms	31 (7.6)	15 (7.5)	7 (7.2)	9 (8.3)	
Multiple symptoms	28 (6.9)	10 (5.0)	7 (7.2)	11 (10.1)	
Co-occurring with pyrexia^a^	91 (22.4)	24 (12.0)	13 (13.4)	54 (49.5)	< 0.001
Co-occurring with urticaria^a^	207 (51.0)	74 (37.0)	57 (58.8)	76 (69.7)	< 0.001
Co-occurring with anaphylactic shock^a^	15 (3.7)	1 (0.5)	5 (5.2)	9 (8.3)	0.002
With personal allergic history^a,b^	152 (37.4)	59 (29.5)	39 (40.2)	54 (49.5)	0.002

*ED* emergency department, *POU* pediatric observation unit

^a^Significant predictors

^b^Asthma, allergic rhinitis

The three most common clinical presentations were respiratory tract symptoms (40.6%), skin lesions (35.2%) and gastrointestinal symptoms (7.6%). The most common duration of angioedema symptoms was 1–2 days (31.8%), followed by 3–4 days (27.1%), less than 1 day (23.9%) and over 4 days (17.2%). The sites of angioedema were presented variously, including face (lip: *n* = 148, 36.6%; eyelid: *n* = 257, 63.6%; cheek: *n* = 62, 15.3%; all: *n* = 56, 13.8%), extremities (upper: *n* = 32, 7.9%; lower: *n* = 23, 5.7%; both: *n* = 36, 8.9%), airway (*n* = 92, 22.7%), bowel (*n* = 24, 5.9%) and trunk (*n* = 15, 3.7%).

Among all the patients, 78 (19.2%) children received oxygen because of respiratory problems. Bag valve mask application and endotracheal intubation were performed in 35 (8.7%) and 8 (2%) children, respectively. None of the children had ever received an emergency tracheostomy or cricothyroidotomy. Twenty-one (5.2%) children were admitted to the PICU, and all of them survived to discharge.

### Factors predicting the severity of the first attack of acute angioedema

The severity of acute angioedema in this study was divided into three groups. In most patients, the angioedema was most commonly mild (directly discharged from the ED, *n* = 200; 49.3%), followed by severe (hospital admission, *n* = 109; 26.8%), and moderate (POU observation, *n* = 97; 23.9%). The factors that might influence the disease severity were analyzed among these three groups (Table 1). Of the patients who developed severe angioedema, 65.1% were male. Most were of preschool age (56.4%), and only 6.4% were adolescents. Severe and moderate angioedema were most commonly caused by various infections and medications, respectively. In the severe angioedema group, most children presented with respiratory tract symptoms. The co-occurrence of pyrexia and the co-occurrence of urticaria were identified more often in the severe angioedema group than in the mild or moderate groups. For all, 71 (17.5%) children have history of asthma, 32 (7.8%) children were co-occurring with asthma attack. Patients with history of asthma (*n* = 50, 70.4%) or co-occurring asthma attack (*n* = 30, 14.6%) were both significantly associate with hospitalization (POU or hospital admission, both *p* < 0.001, using Chi-Square test).

### Factors associated with the duration of the first attack of acute angioedema

#### Etiologies associated with the total duration of acute angioedema

**Fig. 2:**
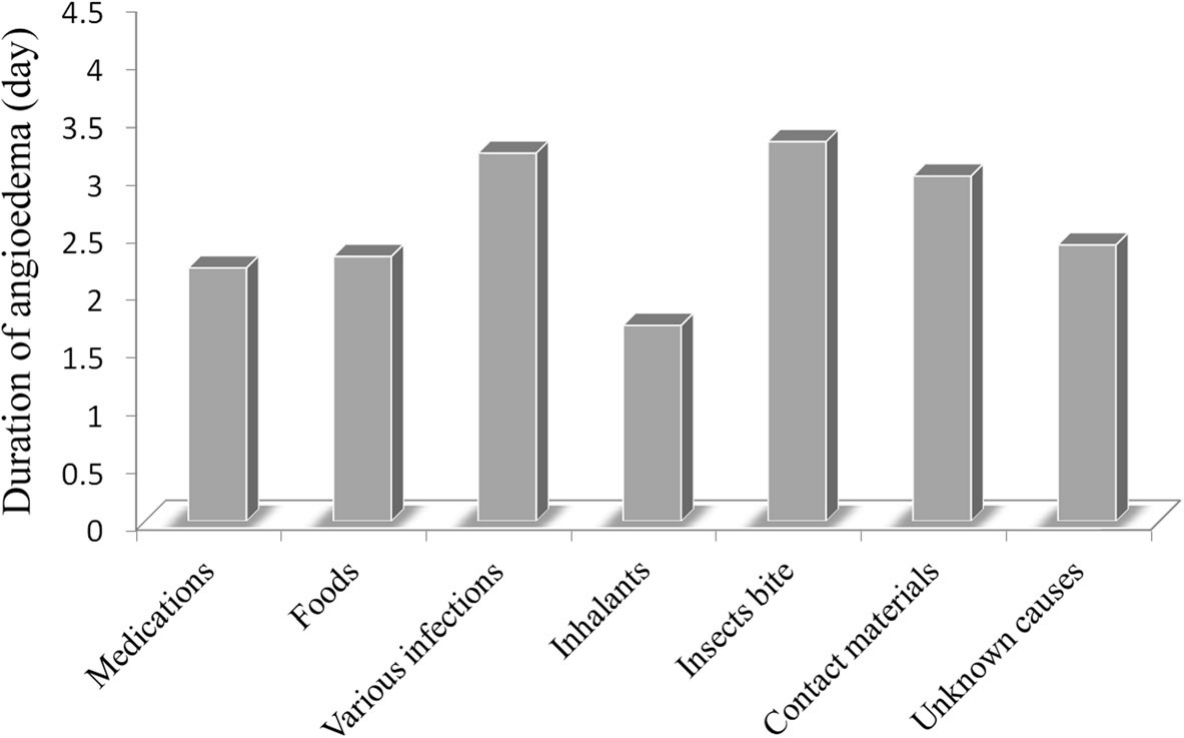
The etiologies significantly associated with the duration of angioedema (*p* < 0.001)

Seven major etiologic categories were identified. These etiologies were then analyzed to determine whether they could predict the duration of acute angioedema. We found that the mean duration of the angioedema differed significantly according to etiology. The longest mean duration of angioedema was associated with insect bites and stings (3.3 ± 1.1 days) and various infections (3.1 ± 1.7 days), followed by contact materials (3.0 ± 0 days), idiopathic causes (2.4 ± 2.2 days), foods (2.3 ± 1.0 days), medications (2.2 ± 1.1 days) and inhalants (1.7 ± 0.8 days) (*P* < 0.001) (Fig. 2).

#### Types of treatments associated with the total duration of acute angioedema

**Fig. 3:**
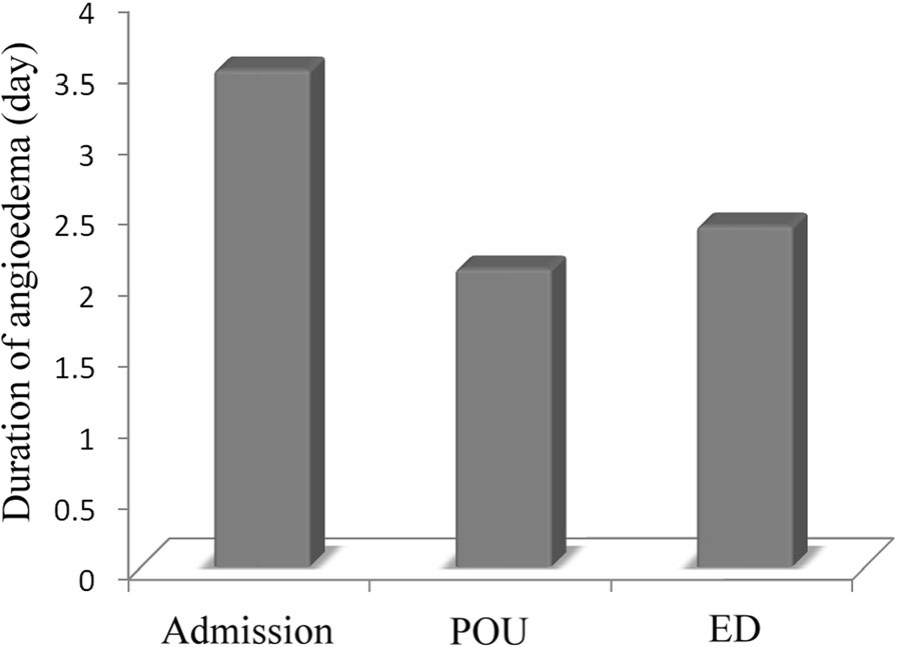
The mean duration of angioedema was shorter in children receiving short-term POU treatments than in those discharged directly from the ED or admitted to the hospital (*p* < 0.001)

**Fig. 4:**
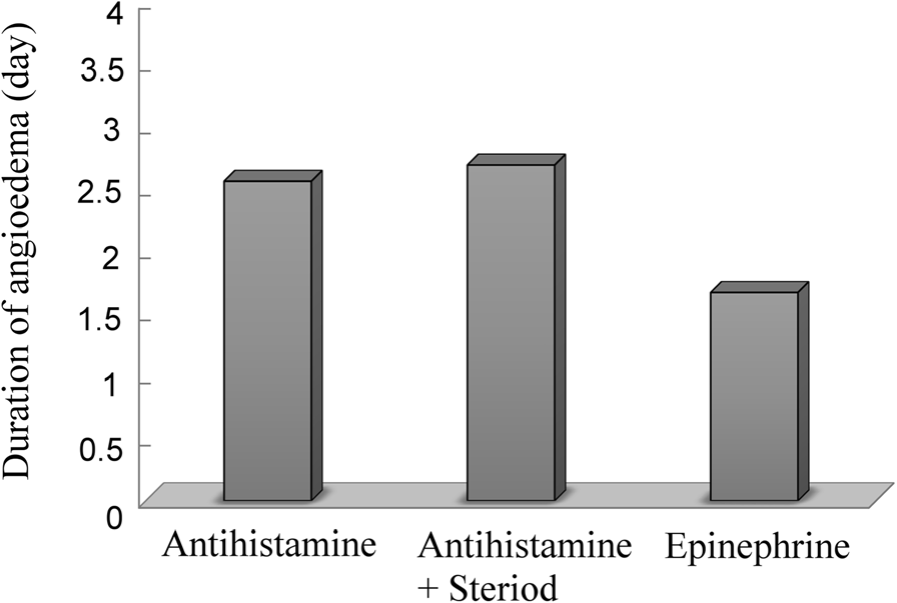
Types of treatments (antihistamines, antihistamine plus corticosteroids and epinephrine) associated with the duration of acute angioedema (*p* < 0.001)

The mean duration of angioedema was 2.3 ± 1.4 days in patients who were discharged from the ED directly (mild angioedema), 2.1 ± 1.1 days in children who required observation in the POU (moderate angioedema), and 3.5 ± 2.0 days in children who were admitted to the hospital (severe angioedema) (*p* < 0.001) (Fig. 3). Antihistamines (*n* = 156, 38.4%), antihistamine plus corticosteroids (*n* = 235, 57.9%) and epinephrine (*n* = 15, 3.7%) were the three major mediations used to treat acute angioedema. The mean duration of angioedema was significantly shorter in children who were administered epinephrine (1.7 ± 1.0 days) than in those who were not administered epinephrine (*p* < 0.001) (Fig. 4).

## Discussion

In this study, we reported the demographics of children with their first attacks of acute angioedema and the detailed possible etiologies. Among the children suffered first attack of angioedema, we found 50.7% of children required hospital stay. Some characteristics of patients would associate more severe course, including co-occurrence of pyrexia or urticaria, etiologies related to medications or infections, presence of respiratory symptoms, and a history of allergies (asthma, allergic rhinitis). Furthermore, we also discussed the influence of treatment on symptom duration. This information could help clinical physicians more effectively treat children suffering from first attacks of angioedema.

### The detailed information of etiologies

Several previous studies reported that milk, egg, and peanut were the most common foods caused allergic/hypersensitive reactions (urticaria, angioedema or anaphylaxis) in children [20, 21]. In this study, seafood was the most common, followed by milk and fruits. Only 6% of foods related angioedema were caused by peanut. Although NSAID and antibiotics were widely thought to be responsible for most medication related angioedema (also 72% in our study) [22, 23]; however, we still noted that 13% of them might be associated with vaccination. Since the causes of hypersensitive reactions of vaccination were usually various and difficult to predict, we suspect post-vaccination observation should not be ignored. Finally, if the allergic history of patient or family is no proof. In relation to food, allergen-specific IgE, skin prick test (SPT) and provocation test might be benefit for patients. For some medications, it is also possible to verify such a suspicion.

### Predictors of severe angioedema

Male sex and preschool age constituted the first set of key predictors of severe angioedema. A previous study reported that the occurrence of angioedema does not differ according to sex or might be slightly more prevalent in adult male patients [24]. A recent study reported that there is a dimorphic sex distribution in the food allergy literature. Among children with food allergies, 64.4% are males and 35.6% are females (male/female ratio, 1.80:1) [25]. However, the relationship between disease severity and sex has not been well addressed. In this study, we found for the first time that male children were not only more likely than female children to develop angioedema but that they were also more likely to suffer severe symptoms. Furthermore, pre-school age children were more associated with severe course (56.9% required hospital admission, *n* = 62). Some previous studies reported that bradykinin-mediated angioedema (hereditary and ACEI related) was rare but more common in young children [26, 27]. However, our population were almost allergic (histamine-mediated) angioedema. Although the allergens tolerance would be developed in most children before school age; we still suggest that allergens exposure should be avoided for pre-school age or younger children [28–30].

The etiologies of infections, foods and medications constituted the second set of key predictors of severe angioedema. Some previous studies reported that ACEI-related angioedema was responsible for 31 to 40% of all angioedema observed in the EDs of the United States and Australia [3, 31]. However, in our study, only 0.5% of patients had angioedema related to ACEI. The major reason for this finding is that ACEI is not the first choice for pediatric hypertension control in Taiwan (due to the side effects of angioedema and coughing). The presence of respiratory symptoms was the third key predictor of severity. Because respiratory tract infections accounted for the majority of infections and might worsen breathing ability (i.e., productive cough, swelling of tonsils), we recommend that airway protection and ventilation (oxygen, endotracheal tube intubation, or even emergency cricothyroidotomy) should be implemented as early as possible [32, 33].

The final set of predictors of the severity of angioedema were a history of allergies and the presence of urticaria. Some angioedema patients could present symptoms of urticaria (mainly wheals on the skin) [2]. In this study, 207 of the children with angioedema (51%) also had urticaria. Previous studies reported that urticaria and angioedema were both caused by reversible vasodilatation and increased vascular permeability, differing only in terms of the tissue depth at which the reaction occurred [34, 35]. Since this study excluded hereditary angioedema and only 2 (0.5%) patients were confirmed as ACEI angioedema; therefore, we suspect our population were almost allergic (histamine-mediated) angioedema. We noted that patients who suffered both urticaria and angioedema, were more likely to require hospital admission. It might be associated with a stronger immune reaction (mast cell/basophil activation was strong enough to trigger both reactions of urticaria and angioedema) or just more uncomfortable of patients [36].

### Predictors of a shorter duration of angioedema

Angioedema caused by infections results in more hospital admissions and a longer duration of symptoms. One previous study reported that acute urticaria caused by infections may indicate a longer clinical course [37]. The major reason might be that the elimination of pathogens (bacteria or viruses) requires a longer course of treatment. To treat angioedema in the ED, physicians should assess and secure the airway. Allergic angioedema should be treated with histamine antagonists and corticosteroids along with epinephrine, as appropriate. Children who received an epinephrine injection had shorter durations of symptoms than those who received only antihistamines or steroids. Patients who needed epinephrine injections often had more severe systemic symptoms, and we found that quick and correct administration of epinephrine clearly shortened the duration of symptoms. Therefore, we suspect that ED physicians should consider using epinephrine as soon as possible in patients with more severe symptoms, especially if anaphylactic shock is co-occurring.

### Recommendation

Finally, we recommend that short-term stays and treatment in the POU might be benefit for patients who did not require hospital admission. The injectable forms of medications (antihistamine and steroid), supplemental fluids and monitoring of vital signs (including the O2 saturation level, allowing the administration of oxygen when needed) could be benefit for angioedema children.

### Limitations

There were some limitations in this study. Some angioedema is non-allergic or hereditary. Non-allergic angioedema has different etiologies and pathogenic mechanisms [38]. Hereditary angioedema is caused by an autosomal dominant genetic mutation. Because this study focused on the first attack of angioedema, patients with hereditary angioedema were not included in this study. The physician’s decision making influenced the classification of severity. For example, most patients who only presented with skin lesions were discharged directly from the ED, while more than half the patients with respiratory tract symptoms required POU stays or hospital admission. In addition, the difference between patients admitted to POU and those sent home might be not clinically significant. It could be influenced by other factors (data on one collected while in observation, the other on follow up, likely days later; different mechanisms of angioedema). Finally, we just compare the duration of angioedema between the three groups, meaning significance could be likely due to the much longer duration in the group of hospital admission.

## Conclusion

The co-occurrence of pyrexia or urticaria, etiologies related to medications or infections, the presence of respiratory symptoms, and a history of allergies were predictors of severe angioedema. More importantly, short-term POU observation and prompt treatment might be benefit for patients who did not require hospital admission.

## Data Availability

Please contact the authors for data requests.

## Abbreviations

ACEI: Angiotensin-Converting Enzyme Inhibitors
ADR: Adverse Drug Reaction
ED: Emergency Department
IRB: Institutional Review Board
OPD: Outpatient Department
POU: Pediatric Observation Unit
SD: Standard Deviation
